# Less Might be More: An Analysis of Energy Intake and Weight Gain in the Treatment of Uncomplicated SAM With RUTF

**DOI:** 10.1111/mcn.70248

**Published:** 2026-10-15

**Authors:** Anura V. Kurpad, Harshpal S. Sachdev

**Affiliations:** ^1^ Department of Physiology St John's Medical College Bengaluru India; ^2^ Department of Paediatrics Sitaram Bhartia Institute of Science and Research New Delhi NCR India

## Abstract

- Children with SAM can be effectively treated with a lower average daily energy intake of 100 kcal/kg/day.
- Their recovery rates are similar to children treated with double (200 kcal/kg/day) this energy intake.

Majer et al. (2026) reported the comparative effectiveness of a Simplified RUTF Treatment Protocol (STP; 2 packets RUTF/day) against a Standard Protocol (SP; 200 kcal/kg/day) for the treatment of uncomplicated SAM. While the recovery with both treatments was the same, a re‐analysis of these data shows that the energy intake of the STP group was actually about 100 kcal/kg/day, supporting the contention that the correct energy requirement for children with SAM should be 105 kcal/kg/day (Sachdev and Kurpad 2024).

In propensity‐score weighted analyses (Table 2), the daily number of sachets consumed by the STP group was lower than the SP group, at 1.4 vs 2.4 sachets/day respectively. However, the weight gain was slightly *greater* in the STP than the SP group (1.6 vs 1.5 kg). This allowed for the calculation of daily weight gain (g/d). From this value, with the reported velocity of weight gain (g/kg/d), the body weight of the children could be calculated at 7 and 6 kg in the STP and SP groups, respectively. From the reported RUTF intake and body weight, the energy intake of the STP group was calculated at 100 kcal/kg/day vs 200 kcal/kg/day in the SP group. Both intakes were equally effective (85.6% vs 86.4% recovery respectively).

The slightly longer duration of recovery in the STP group (~5 days or 10% duration) is probably a price worth paying, considering the risk of greater fat deposition and a later double burden of malnutrition (Sachdev et al. 2021). Future body compositional studies would be informative.

## Author Contributions

A.V.K. and H.P.S. did the analyses and wrote this letter. No AIGC tools were used in developing any part of this letter.

## Conflicts of Interest

The authors declare no conflicts of interest.

## Data Availability

The authors have nothing to report.
